# Long-Term Pancreatic Functional Impairment after Surgery for Neuroendocrine Neoplasms

**DOI:** 10.3390/jcm8101611

**Published:** 2019-10-03

**Authors:** Valentina Andreasi, Stefano Partelli, Gabriele Capurso, Francesca Muffatti, Gianpaolo Balzano, Stefano Crippa, Massimo Falconi

**Affiliations:** 1 Pancreatic Surgery Unit, Pancreas Translational & Clinical Research Center, San Raffaele Scientific Institute IRCCS, 20132 Milan, Italy; andreasi.valentina@hsr.it (V.A.); partelli.stefano@hsr.it (S.P.); muffatti.francesca@hsr.it (F.M.); balzano.gianpaolo@hsr.it (G.B.); crippa1.stefano@hsr.it (S.C.); 2 Faculty of Medicine and Surgery, “Vita-Salute San Raffaele” University, 20132 Milan, Italy; 3 Biliopancreatic Endoscopy Unit, Pancreas Translational & Clinical Research Center, San Raffaele Scientific Institute IRCCS, 20132 Milan, Italy; capurso.gabriele@hsr.it

**Keywords:** pancreatic neuroendocrine neoplasms, neuroendocrine tumor, long-term functional outcomes, pancreatectomy, diabetes mellitus, pancreatic exocrine insufficiency, body mass index, parenchyma-sparing surgery

## Abstract

Radical surgery represents the only curative treatment for pancreatic neuroendocrine neoplasms (PanNEN). The aim of this study was to evaluate the postoperative onset of diabetes mellitus (DM) and/or pancreatic exocrine insufficiency (PEI) in surgically treated PanNEN. Consecutive PanNEN patients, without preoperative DM, who underwent partial pancreatic resection, were included. After a median follow-up of 72 months, overall 68/276 patients (24%) developed DM. Patients who developed DM were significantly older (*p* = 0.002) and they had a higher body mass index (BMI) (*p* < 0.0001) than those who did not; they were more frequently male (*p* = 0.017) and with nonfunctioning neoplasms (*p* = 0.019). BMI > 25 Kg/m^2^ was the only independent predictor of DM (*p* = 0.001). Overall, 118/276 patients (43%) developed a PEI, which was significantly more frequent after pancreaticoduodenectomy (*p* < 0.0001) and in patients with T3-T4 tumors (*p* = 0.001). Pancreaticoduodenectomy was the only independent predictor of PEI (*p* < 0.0001). Overall, 54 patients (20%) developed disease progression. Patients with and without DM had similar progression free survival (PFS), whereas patients without PEI had better five-year-PFS (*p* = 0.002), although this association was not confirmed in multivariate analysis. The risk of DM and PEI after surgery for PanNEN is relatively high but it does not affect PFS. BMI and pancreatic head resection are independent predictors of DM and PEI, respectively.

## 1. Introduction

Pancreatic neuroendocrine neoplasms (PanNEN) represent less than 3% of all pancreatic lesions. Despite being still considered rare tumors, their incidence has dramatically increased during the last two decades, which is probably due to the widespread use of high-quality imaging techniques [1,2]. PanNEN exhibit heterogeneous biological behaviour, which ranges from indolent to aggressive forms. Overall, the survival rates for PanNEN are better than those that were reported for their exocrine counterpart. The vast majority of PanNEN is represented by well-differentiated forms (PanNET) with a reported five-year survival rate of 70–90% for patients with localized PanNEN that decreases to 40–60% for patients with metastatic disease [3,4,5]. Radical surgery represents the backbone for the curative treatment of PanNEN [6]. Therefore, given the good prognosis and the high rate of cure of these neoplasms, it is of paramount importance to carefully weigh the oncological risk along with the long-term functional outcomes following pancreatic resection. In particular, the onset of diabetes mellitus (DM) and/or pancreatic exocrine insufficiency (PEI) might have a considerable impact on the general health status and on the quality of life of these patients [7,8]. At this regard, it has been reported that malnutrition that results from PEI can lead to the development of comorbidities that negatively impact on prognosis [9,10]. For these reasons, parenchyma-sparing surgical procedures (i.e., enucleation and middle pancreatectomy) have been proposed for reducing the incidence of postoperative pancreatic endocrine and exocrine insufficiency [11,12,13,14] and it has been widely reported that parenchyma-sparing surgery, enucleation in particular [15,16], is associated to improved long-term functional outcomes as compared to formal resections [11,16,17,18,19]. The likelihood of developing pancreatic insufficiency depends on the extent of pancreatic resection as well as on the functionality of the remaining parenchyma [19]. Several studies, investigating patients with different benign or low-grade malignant lesions, have shown that the type of surgical procedure (parenchyma-sparing vs. standard resection), but also other patients’ related factors, such as age or the presence of chronic pancreatitis, might contribute to the post-surgical development of pancreatic insufficiency [19,20,21].

Aim of the present study was to evaluate the rate of long-term pancreatic impairment, defined as postoperative onset of DM and/or of PEI, in a series of patients submitted to partial pancreatic resection for PanNEN and investigate factors that are associated with it. The secondary aim was to evaluate a possible effect of pancreatic insufficiency on progression free survival (PFS).

## 2. Experimental Section

### 2.1. Study Design

**Figure 1:**
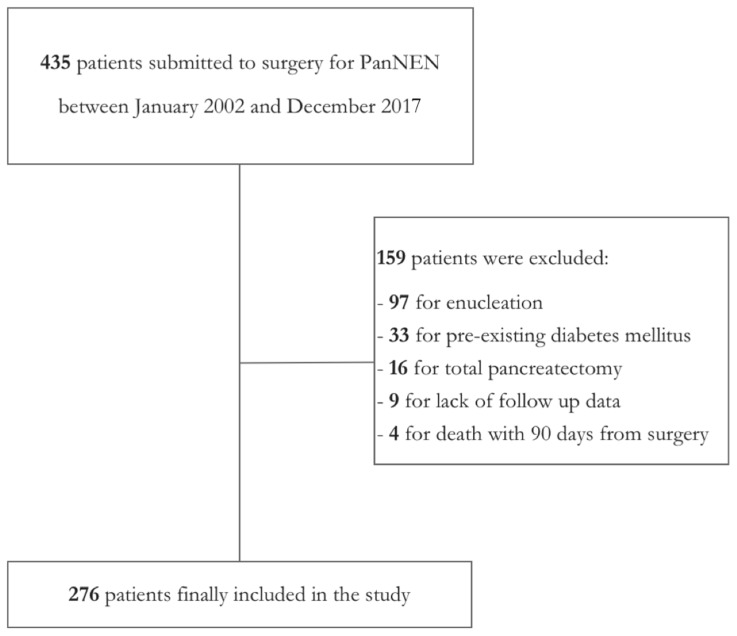
Flowchart representing patients included in the study.

This retrospective cohort study was conducted following the Strengthening the Reporting of Observational Studies in Epidemiology Statement (STROBE) guidelines [22]. All of the patients who underwent surgery for PanNEN at San Raffaele Scientific Institute (Milan) between January 2002 and December 2017 were retrospectively screened. Patients submitted to partial pancreatic resection (pancreaticoduodenectomy (PD), distal pancreatectomy (DP), atypical resection (AR)) for PanNEN with available long-term functional outcomes data were included in the study. Patients with a preoperative diagnosis of DM and/or PEI as well as those who underwent total pancreatectomy for PanNEN, were excluded from the present study. Patients submitted to enucleation were also excluded, as this surgical procedure, which consisted in the removal of just the tumor without resecting pancreatic parenchyma, could not be considered as a partial pancreatic resection. Patients who deceased within 90 days from operation due to surgical complications were also excluded. Figure 1 depicts the initial number of patients who were screened and the final study population.

### 2.2. Definition of Outcomes

The postoperative onset of DM and PEI represented the main outcome of this study. According to the American Diabetes Association diagnostic criteria [23], postoperative DM was defined when glycated haemoglobin (HbA1c) was equal to or greater than 6.5% and/or in the presence of a fasting plasma glucose (FPG) equal to or greater than 126 mg/dL and/or in a patient with classic symptoms of hyperglycemia or hyperglycemic crisis associated to a random plasma glucose that was equal to or greater than 200 mg/dL. FPG and HbA1c were measured in all of the patients before surgery. PEI was diagnosed in the presence of manifest clinical signs of malabsorption and/or maldigestion (steatorrhea, weight loss, flatulence, and abdominal distention), which improved with the assumption of pancreatic enzyme replacement therapy. The secondary outcome of this study was represented by the possible effect of pancreatic insufficiency on PFS.

All of the patients were followed up regularly after surgery. High-quality imaging examination, as well as blood tests inclusive of HbA1c and FPG, were performed at least every six months (in the absence of signs or symptoms of hyperglycemia). A follow-up phone call was scheduled on a six-month basis, whereas an outpatient visit was planned on a yearly basis. Information regarding general health status and possible signs or symptoms of pancreatic insufficiency was collected during outpatient visits or by telephone. Last follow up was updated in June 2019. Progression free survival (PFS) was defined as the time from surgery to the first evidence of disease recurrence or progression and it was censored at the last follow up if no disease relapse had occurred. Overall survival (OS) was defined as the time from surgery to death and censored at the last follow up if no events had happened.

### 2.3. Data Collection

Demographic data, perioperative details, and pathological findings were retrospectively retrieved from an electronic database. Preoperative variables considered were: age, gender, body mass index (BMI), tumor functionality, and the presence of an inherited syndrome. The choice of the surgical technique was based on the location, the size, and the preoperative aggressiveness features of the neoplasm. PD and DP were routinely performed for tumors that were located in the head and in the body-tail of the pancreas, respectively. Middle pancreatectomy was performed in the presence of small tumors < 4 cm, which could not be removed by enucleation, located in the pancreatic neck/proximal body and without features of aggressiveness. Middle-preserving pancreatectomy was chosen in the presence of a multifocal disease (i.e., multiple endocrine neoplasia (MEN) type 1) involving pancreatic head and tail, but skipping the body of the gland [24]. Islet autotransplantation was carried out, although not routinely, in patients requiring a DP for a benign/borderline PanNEN located in the pancreatic body/neck [25]. This procedure started being performed in January 2009 and it is still ongoing. The Clavien-Dindo classification system was used to assess the severity of postoperative complications [26]. The rates of postoperative pancreatic fistula (POPF) [27], abdominal collection, hemoperitoneum, blood transfusion, and readmission were evaluated. Length of hospital stay (LOS) and operative time were also considered. At final histology, PanNEN were classified according to the current TNM European NeuroEndocrine Tumor Society (ENETS) classification [28]. Ki67 proliferative index was assessed in the surgical specimen by MIB1 antibody staining and evaluated by measuring the percentage of cells with positive nuclear staining after the count of 2000 cells in the area of highest nuclear labelling [3]. Tumor grade was defined according to the 2017 World Health organization (WHO) Classification [3].

### 2.4. Statistical Analysis

Continuous variables were expressed as mean and standard deviation (SD) for normally distributed data and as median and interquartile range (IQR) for skewed distributions. The categorical variables were presented as numbers and percentages (%). The comparison between subgroups was performed using Student’s *t* test or Mann-Whitney U test, for continuous variables as appropriate. Qualitative data were compared by the Chi square test or Fisher’s exact test, when appropriate. Multivariate logistic regression analysis was performed to evaluate the predictors of postoperative DM and of PEI. Survival probability was estimated according to the Kaplan-Meier method. Multivariate analysis to evaluate significant predictors of PFS was performed by the Cox regression model. Follow-up was updated on June 2019, giving a potential minimum follow-up of 18 months to each patient. Statistical analyses were performed in SPSS 25.0 for Mac (SPSS Inc., Chicago, IL, USA). *p* values were considered to be significant when less or equal than 0.05.

## 3. Results

### 3.1. Study Population

Overall, 276 patients were included in the present study. Of these, 76 patients (27%) underwent PD, whereas 192 (70%) were submitted to DP. Atypical parenchyma-sparing resections were performed in the remaining eight cases (3%) (*n* = 7 middle pancreatectomy, *n* = 1 middle-preserving pancreatectomy). Table 1 summarizes perioperative details.

### 3.2. Postoperative DM

At a median follow-up of 72 months (IQR 38;103 months) after surgery, 68 patients (24%) developed a postoperative DM. Table 2 reports a comparison of demographic, perioperative and pathological characteristics between patients who developed DM and those who did not. Patients who developed DM were significantly older when compared to those who did not develop DM (median 60 years (IQR 56;67 years) vs. 56 years (IQR 46;67 years), *p* = 0.002). The median preoperative BMI was significantly higher in patients who developed postoperative diabetes (median 27 Kg/m^2^ (25;30 Kg/m^2^) vs. 24 Kg/m^2^ (IQR 22;27 Kg/m^2^), *p* < 0.0001). Postoperative DM presented more frequently in males than in females (*p* = 0.017), as well as in patients that were diagnosed with nonfunctioning neoplasms as compared to patients with functioning tumors (*p* = 0.019). In the group of patients who developed DM, functioning PanNEN (*n* = 6) were insulinomas in five cases (83%) and a VIPoma in one case. The rate of postoperative diabetes was similar between patients submitted to different surgical procedures (*p* = 0.476). Among those eight patients (3%) submitted to an AR, the onset of DM was observed in two cases after middle pancreatectomy. No differences were found in terms of DM rate between patients who developed high-grade vs. low-grade or no postoperative complications (*p* = 0.647). Among those nine patients who underwent islet autotransplatation, the onset of DM was observed in four cases. All these four patients had a BMI greater than 25 Kg/m^2^ (in three out of four cases BMI was greater than 30 Kg/m^2^). None of the patients submitted to islet autotransplantation developed complications related to the procedure. At multivariate logistic regression analysis (Table 3), a BMI that was greater than 25 Kg/m^2^ was the only independent predictor of postoperative DM (Odds Ratio (OR) 4.945, 95% Confidence Interval (C.I.) 1.889–12.943, *p* = 0.001). The rates of DM in normal-weight, overweight, and obese patients were 8%, 32%, and 38%, respectively. Among male patients with a BMI greater than 25 Kg/m^2^, the development of postoperative DM was observed in 40% of cases. This rate increased to 50% when the study population was stratified while using 28 Kg/m^2^ as BMI cut-off.

### 3.3. Postoperative PEI

Overall, 118 patients (43%) developed a postoperative PEI. Table 4 reports a comparison of demographic, perioperative, and pathological characteristics between patients who developed PEI and those who did not. The onset of PEI was significantly more frequent after PD when compared to DP and atypical resections (*p* < 0.0001) as well as in patients that were diagnosed with T3–T4 tumors as compared to patients with T1–T2 tumors (*p* = 0.001). Among the eight patients (3%) who underwent an AR, the appearance of postoperative PEI was observed in two cases (*n* = 1 middle pancreatectomy, *n* = 1 middle-preserving pancreatectomy). Median preoperative BMI in patients with a diagnosis of postoperative PEI was 24 Kg/m^2^ (IQR 22;25 Kg/m^2^) as compared to 25 Kg/m^2^ (IQR 23;28 Kg/m^2^) (*p* = 0.005). Male patients with a BMI that was greater than 25 Kg/m^2^ developed PEI in 20% of cases. Patients who developed high-grade postoperative complications (Clavien-Dindo III-IV) displayed a higher frequency of PEI (*p* = 0.027). At multivariate logistic regression analysis (Table 5) pancreaticoduodenectomy was the only independent predictor of postoperative pancreatic exocrine insufficiency onset (OR 31.680; 95% CI 10.622–94.487; *p* < 0.0001).

### 3.4. Long-Term Oncological Outcomes

After a median follow-up of 72 months (IQR 38;103 months), 54 patients (20%) developed a disease recurrence, and 22 (8%) eventually died of disease. Overall, 11 patients (4%) died for other causes that were not tumor-related. The overall PFS and OS rates at five years were 80% and 91%, respectively. The effect of postoperative DM and PEI was then tested against PFS, whereas it was not tested against OS, since the number of disease-specific deaths was too low. No statistically significant differences were found in terms of PFS between patients with and without postoperative DM (five-year PFS rate 80% vs. 80%, *p* = 0.827). Patients without PEI had better PFS when compared to patients who developed a postoperative PEI (five-year PFS rate 86% vs. 71%, *p* = 0.002). At multivariate analysis, adjusted for age, gender, T stage, N stage, M stage, grading, microvascular invasion, perineural invasion, and necrosis, postoperative exocrine pancreatic insufficiency was no longer a predictor of disease recurrence/progression (Hazard Ratio (HR) 1.497; 95% CI 0.840–2.669; *p* = 0.171).

## 4. Discussion

PanNEN have a more indolent biological behaviour and they are usually associated to a longer survival when compared to their exocrine counterpart. Therefore, it is of paramount importance to evaluate the long-term functional sequelae following pancreatic resection for PanNEN and to find a balance between the oncological risk and the impact of endocrine and exocrine impairment on general health status. Various studies have explored the functional outcomes after pancreatic resection in large populations, including patients affected by different pancreatic diseases, ranging from benign conditions to cancer [19,20,29,30,31]. In contrast, data on the long-term endocrine and exocrine pancreatic insufficiency after pancreatic surgery specifically performed for PanNEN are currently limited [32]. The risk of developing a postoperative DM and/or PEI can be influenced by specific characteristics that are related to the underlying primary pancreatic disease.

The incidence of post-pancreatectomy DM ranges from 5% to 78% [20,30,31,33,34], probably due to the heterogeneity of the selecting criteria of study populations and to the different duration of follow-up. In the present series, the onset of postoperative DM was observed in nearly one-third of patients after six years from surgery. A similar incidence of DM (23%) was reported in a series including 229 patients submitted to surgery for benign tumors [20]. In contrast, the incidence reported by Falconi et al. [19] in a previous study including only benign diseases was lower, with postoperative DM being reported only in the 14% of cases after DP and in the 18% of cases after PD, respectively [19]. Similarly, another series, including only benign or low-grade malignant neoplasms, reported a low incidence of postoperative DM (<10%) after a median follow-up of less than two years [35]. The higher incidence of DM found in the present series is probably related to the longer duration of follow-up, which also represents one of the main strengths of the present study. Various factors have been described as being able to influence the risk of developing endocrine insufficiency: these include the extent of resection, the nature of disease, some patient’s characteristics, and the functionality of the remaining parenchyma [19].

In the present series, BMI was found to be the only independent predictor of postoperative DM: specifically, a BMI greater than 25 Kg/m^2^ increased the risk of developing postoperative DM up to five times. Of note, four out of nine patients submitted to islet autotransplantation developed postoperative DM: all of them had a BMI greater than 25 Kg/m^2^. This result corroborates previous findings reporting that increasing BMI is associated to a higher risk of postoperative endocrine insufficiency [20,29,36]. This result confirms the importance of a personalised prehabilitation before surgery in those patients who are overweight or obese. At this regard, the relatively indolent nature of PanNEN allows for safely postponing the day of operation from initial diagnosis. The result here presented is consistent with data from the National Health and Nutrition Examination Surgery (NAHNES) reporting that the prevalence of DM in general population increases with the increasing of BMI class [37]. According to this survey, the prevalence of DM among normal-weight patients is around 8%, whereas it is reported to almost double (15%) in the overweight patients. The prevalence of DM increases even more in obese patients, attesting itself around 28% [37]. In the present series, overweight patients developed postoperative DM in 32% of cases (vs. 15% in general population), whereas the rate of DM among obese patients was 38% (vs. 28% in general population). In contrast, normal-weight patients developed postoperative DM in 8% of cases, consistently with data that were reported in general population. Moreover, patients who developed a postoperative DM were more frequently males and had an older age compared to those who did not. Although these findings were not confirmed at multivariate analysis, they represent well-known risk factors for DM and they were also reported by other series as factors that are associated to the development of postoperative DM [19,29]. In particular, according to data from the Study to Help Improve Early evaluation and management of risk factors Leading to Diabetes (SHIELD), male patients with a high BMI (≥ 28 Kg/m^2^) display a DM prevalence of around 40%, whereas in the present series half of patients with the same characteristics developed DM, which suggested that the pancreatic resection has a role in determining the onset of the disease. Interestingly, no statistically significant differences were found in terms of risk of developing DM between patients submitted to different surgical procedures, even if a trend towards a higher incidence of DM after DP (26%) than after PD (20%) was observed, as previously reported by other series [20,29]. Probably, in the present series, the difference between DP and PD failed to reach a statistically significant difference because patients that were submitted to DP had smaller tumors when compared to patients who underwent PD. Consequently, the extent of DP was often limited for sparing parenchyma and preserving its functionality. Various studies have previously reported a lower incidence of postoperative pancreatic impairment after parenchyma-sparing surgery [15,17,18,19]. In the present series, patients that were submitted to enucleation were excluded in order to focus on partial pancreatic resections; therefore, as only eight patients submitted to atypical resections (middle pancreatectomy or middle-preserving pancreatectomy) were considered, a statistically significant difference in terms of DM development between these subjects and those that were submitted to a formal resection could not be demonstrated. However, when patients also submitted to enucleation were considered for this specific analysis, the rate of postoperative DM was significantly lower (*p* = 0.001) in those that were submitted to a parenchyma-sparing surgery (10%) when compared to those who underwent a formal resection (25%).

The occurrence of PEI is another important outcome following pancreatic resection [7]. PEI is frequently misdiagnosed, as it usually presents with mild or moderate symptoms that may be underestimated, leading to a poor quality of life [8], micronutrients deficiencies [38] and decreased survival [10]. In the present study, the overall incidence of PEI was 43% that is consistent with the rate reported by Lim et al. [39]. The rate of PEI development that was reported in literature varies between 56% and 98% after PD [7,8,40] and between 19% and 80% after DP [7]. This wide range is probably due to the different methods that were used to assess pancreatic exocrine function and to the low accuracy of available tests in determining PEI [41]. Of note, in the present series, exocrine impairment was observed in nearly nine out of 10 patients after PD and this operation was found to be independently associated with an increased risk of PEI. PD has been widely demonstrated to be strongly correlated to PEI [29,39,42]. The higher frequency of PEI after PD is essentially explained by the surgical reconstruction, as it can predispose to a progressive damage of the remaining pancreatic stump [43], to bile salt malabsorption [44] and to bacterial overgrowth [7]. In the present series, a lower rate of PEI among patients submitted to atypical resection could not be demonstrated, as only eight patients undergoing this kind of surgery were included. However, when also patients submitted to enucleation were considered for this specific analysis, the rate of PEI after parenchyma-sparing surgery was significantly lower (2%) than after formal resection (43%). Moreover, a lower preoperative BMI was found to be associated with a higher rate of PEI, as previously reported by Kusakabe et al. [29]. At this regard, it is possible that patients with a lower preoperative BMI have an undiagnosed preoperative PEI and, consequently, they are more likely to develop an evident PEI after pancreatic resection. Finally, patients who developed high-grade postoperative complications displayed a significantly higher rate of PEI when compared to other patients. However, this association was not confirmed at multivariate analysis, probably because patients with high-grade postoperative complications were the same who underwent PD, which is an independent predictor of PEI development.

Our findings are in partial agreement with the few previous reports that were obtained in smaller series. Neophytou et al. [32] investigated the postoperative rate of DM and PEI in 92 patients operated for benign tumours, including PanNEN. Factors that were associated with the occurrence of DM were male sex, a BMI > 28 Kg/m^2^ and metabolic syndrome, whereas factors that were associated with the risk of PEI were preoperative chronic pancreatitis, a BMI < 18.5 Kg/m^2^ and tumors located in the pancreatic head. Of note, although the role of chronic pancreatitis in the remnant pancreas was not investigated, this is unlikely to be relevant in PanNEN, as patients who undergo pancreatic resection for these neoplasms usually have a normal, non-fibrotic, pancreatic remnant that was not affected by the presence of the tumor. Indeed, PanNEN typically exhibit an expansive evolution rather than an infiltrative growth.

In the present series, DM occurred as a gradual phenomenon, as the majority of patients did not develop it immediately after surgery, but during follow up, over the course of several months or even years, consistently with data that were previously reported by Falconi et al. [19]. This finding corroborates the fact that the development of DM is not only dependent from the surgical procedure, but even after a pancreatic resection, other factors, such as a BMI > 25 Kg/m^2^, strongly contribute to its appearance. In contrast, most of patients developed PEI in the early postoperative period, probably because its occurrence is strictly related to the surgical procedure. As previously pointed out, PD is more frequently associated with PEI and its early occurrence might be related not only to the reduced pancreatic volume, but also to a sudden impairment of pancreatic stimulation, which is physiologically induced by endocrine cells of the resected duodenum [43]. However, one could speculate that patients that were submitted to PD could experience a worsening of PEI during follow up as the surgical reconstruction associated to PD can predispose to progressive damage and atrophy of the pancreatic stump.

The secondary outcome of the present study was to investigate whether endocrine or exocrine pancreatic insufficiency were associated with disease outcome. We focused on the association with PFS, as the rate of disease-related deaths was low, as expected for surgically treated PanNEN. While DM was not associated with PFS, there was a lower five-year PFS rate in patients who developed PEI. However, when corrected for other prognostic factors at multivariate regression, PEI was not a significant factor.

The overall rate of postoperative DM and PEI observed in the present series is relatively high (24% and 43% for DM and PEI, respectively), and it has been reported that pancreatic impairment might be associated with a significant impact on general health status and on quality of life [7,8]. This is one of the reasons in support of an active surveillance management instead of a pancreatic resection for patients that were affected by non-functioning PanNEN ≤ 2 cm without features of aggressiveness [6,45,46].

The present study has several limitations. The major limit is represented by the retrospective design. Secondly, the diagnosis of PEI was not based on specific tests objectively evaluating the pancreatic function, but on the presence of related signs and symptoms that were cured with pancreatic enzymes replacement treatment. However, the accuracy and feasibility of the available tests are currently debated [41]. Indirect tests, such as fecal elastase-1, fecal chymotrypsin, and 13C breath test, evaluate the quantitative changes of pancreatic secretion and are less expensive, easier to be performed, but less accurate, compared to direct ones. Direct tests, on the contrary, evaluate directly the secretive production, but, despite their good sensitivity, are invasive, time-consuming, and expensive [41]. However, the use of both these test after pancreatic surgery is unreliable. Indeed, it has been reported that fecal elastase 1 is not accurate in diagnosing PEI after pancreatic surgery [47]. 13C breath test has been previously performed to evaluate pancreatic exocrine function in patients that were submitted to pancreatic resection [48,49] and it seems to be more accurate than fecal elastase-1 [48]. However, the validity of 13C breath test is still questionable as a comparison between this test and a gold standard (72 h fecal fats or bicarbonate dosage in pancreatic juice) in patients that were submitted to pancreatic surgery has not been made. Of note, when PD is performed, besides the reduced enzyme output following the removal of pancreatic parenchyma, other factors, such as small bowel bacterial overgrowth, deranged antral grinding, abnormal mixing of food with digestive secretions, abnormal hormonal stimulation, and acidic intraluminal pH, can affect the results [47]. Moreover, various steps, including gastric emptying time of the tracer, absorption, hepatic circulation, and metabolism, are involved in breath test and some of them might be altered after pancreatic resection [48]. Regarding direct tests, such as endoscopic aspiration of pancreatic juice, it has to be said that they are invasive and cannot be performed when anatomy is modified by surgical procedures [40]. Another possible limitation of the present study is represented by the lack of data on the possible role of medical treatments initiated during follow-up for a recurrence of the PanNET, which might have contributed to occurrence of PEI [50]. However, the rate of PEI occurring after tumor recurrence was 54% in patients that were treated with somatostatin analogues and 69% in patients who did not us them, which suggests that this is not a relevant issue. Finally, a more complete analysis of pancreatic endocrine function with the execution of oral glucose tolerance test (OGTT), dosage of insulin and C-peptide, and calculation of Homeostatic Model Assessment for Insulin Resistance (HOMA-IR) could have been performed, thus adding interesting information regarding glucose metabolism in patients that were submitted to pancreatic resection. However, according to the current American Diabetes Association (ADA) guidelines, either fasting plasma glucose (FPG), 2-h plasma glucose during 75 g OGTT and HbA1c are equally appropriate for diagnosing DM [23]. In particular, HbA1c seems to have some advantages when compared to both FPG and OGTT, as it is reported to have a greater convenience (as fasting is not required), a greater pre-analytical stability, and fewer perturbations during stress and illness [23]. This is an important point given the fact that patients who undergo a pancreatic resection are subjected to a severe physical stress, which could easily alter plasma glucose levels.

In conclusion, the present study demonstrated that the risk of postoperative pancreatic endocrine and exocrine insufficiency after surgery for PanNEN is significantly high and patients should be aware of these complications. A personalized prehabilitation should be recommended in those patients with a BMI > 25 kg/m^2^ for reducing the risk of DM development in the postoperative period. Endocrine and exocrine insufficiency do not seem to influence PFS. Further studies are needed to better elucidate the time of onset and the severity of DM and/or PEI and to assess their impact on quality of life of patients that were surgically treated for PanNEN.

## Figures and Tables

**Table 1:** Perioperative details of 276 patients submitted to surgery for pancreatic neuroendocrine neoplasms (PanNEN).

Variable	*n* (%)
**Operative time**, min ^1^	240 (180;300)
**Length of stay**, days ^1^	9 (7;11)
**Readmission**	
No	242 (88)
Yes	34 (12)
**Blood transfusion**	
No	229 (83)
Yes	47 (17)
**Islet autotransplatation**	
No	267 (97)
Yes	9 (3)
**Complications** [26]	
No complications	94 (34)
I	56 (20)
II	89 (32)
III	36 (13)
IV	1 (1)
**POPF [27]**	
No	147 (53)
Yes	129 (47)
**Abdominal Collection**	
No	223 (81)
Yes	53 (19)
**Postoperative Hemorrhage**	
No	262 (95)
Yes	14 (5)

^1^ Expressed as median [interquartile range (IQR)]. POPF: Postoperative Pancreatic Fistula.

**Table 2:** Comparison of demographic, clinical and pathological characteristics between patients submitted to surgery for pancreatic neuroendocrine neoplasms (PanNEN) who developed postoperative diabetes mellitus (DM) (*n* = 68) and those who did not (*n* = 208).

Variable	Total Population	No Postoperative DM	Postoperative DM	*p* Value
	*n* = 276	*n* = 210	*n* = 68	
**Age**, years	58 (49;67)	56 (46;67)	60 (56;67)	**0.002**
**Gender**				
Male	138 (50)	95 (46)	43 (63)	
Female	138 (50)	113 (54)	25 (37)	**0.017**
**Preoperative BMI**, Kg/m^2^	25 (22;27)	24 (22;27)	27 (25;30)	**<0.0001**
**PanNEN functionality**				
Nonfunctioning	225 (82)	163 (78)	62 (91)	
Functioning	51 (18)	45 (22)	6 (9)	**0.019**
**Inherited Syndrome**				
No	261 (95)	194 (93)	67 (99)	
Yes	15 (5)	14 (7)	1 (1)	0.127
**Type of Surgery**				
Pancreaticoduodenectomy	76 (27)	61 (29)	15 (22)	
Distal Pancreatectomy	192 (70)	141 (68)	51 (75)	
Atypical Resection	8 (3)	6 (3)	2 (3)	0.476
**T stage** [28]				
T1–T2	180 (65)	136 (65)	44 (65)	
T3–T4	96 (35)	72 (35)	24 (35)	0.919
**Tumor grade** [3]				
G1	153 (55)	110 (53)	43 (63)	
G2	110 (40)	85 (41)	25 (37)	
G3	13 (5)	13 (6)	0 (0)	0.065
**Complications** [26]				
No-I-II	239 (87)	179 (85)	60 (88)	
III-IV	37 (13)	29 (15)	8 (12)	0.647

BMI: Body Mass Index; PanNEN: Pancreatic Neuroendocrine Neoplasm; Data are expressed as number (%) or interquartile range (IQR).

**Table 3:** Multivariate logistic regression analysis of predictors of postoperative diabetes mellitus (DM).

Variable	OR	95% C.I.	*p*
**Gender**			
Male	1	-	
Female	0.481	0.178–1.305	0.151
**Age**			
≤60 years	1	-	
>60 years	0.972	0.366–2.579	0.954
**Preoperative BMI**			
≤25 Kg/m^2^	1	-	
>25 Kg/m^2^	4.945	1.889–12.943	**0.001**
**Type of PanNEN**			
Nonfunctioning	1	-	
Functioning	0.269	0.071–1.022	0.054

BMI: Body Mass Index; PanNEN: Pancreatic Neuroendocrine Neoplasm.

**Table 4:** Comparison of demographic, clinical and pathological characteristics between patients submitted to surgery for pancreatic neuroendocrine neoplasms (PanNEN) who developed postoperative pancreatic exocrine insufficiency (PEI) (n = 118) and those who did not (n = 158).

Variable	Total Population	No Postoperative PEI	Postoperative PEI	*p* Value
	*n* = 276	*n* = 158	*n* = 118	
**Age**, years	58 (49;67)	58 (49;65)	60 (47;68)	0.556
**Gender**				
Male	138 (50)	76 (48)	62 (53)	
Female	138 (50)	82 (52)	56 (47)	0.543
**BMI**, Kg/m^2^	24.5 (22.5;27)	25 (23;28)	24 (22;25)	**0.005**
**Type of PanNEN**				
Non-functioning	225 (82)	123 (78)	102 (86)	
Functioning	51 (18)	35 (22)	16 (14)	0.085
**Inherited Syndrome**				
No	261 (95)	150 (95)	111 (94)	
Yes	15 (5)	8 (5)	7 (6)	0.793
**Type of Surgery**				
Pancreaticoduodenectomy	76 (27)	8 (5)	68 (58)	
Distal Pancreatectomy	192 (70)	144 (91)	48 (41)	
Atypical Resection	8 (3)	6 (4)	2 (1)	**<0.0001**
**T stage** [28]				
T1–T2	180 (65)	116 (73)	64 (54)	
T3–T4	96 (35)	42 (27)	54 (46)	**0.001**
**Tumor grade** [3]				
G1	153 (55)	96 (61)	57 (48)	
G2	110 (40)	55 (35)	55 (47)	
G3	13 (5)	7 (4)	6 (5)	0.108
**Complications** [26]				
No-I-II	239 (87)	143 (91)	96 (81)	
III-IV	37 (13)	15 (9)	22 (19)	**0.027**

BMI: Body Mass Index; PanNEN: Pancreatic Neuroendocrine neoplasm; Data are expressed as number (%) or median (interquartile range (IQR)).

**Table 5:** Multivariate logistic regression analysis of predictors of postoperative pancreatic exocrine insufficiency.

Variable	OR	95% C.I.	*p*
**BMI**			
≤25 Kg/m^2^	1	-	
>25 Kg/m^2^	0.746	0.280–1.989	0.558
**Type of Surgery**			
Distal Pancreatectomy	1	-	
Pancreaticoduodenectomy	31.68	10.622–94.487	**<0.0001**
Atypical resection	4.8	0.626–36.818	0.131
**T stage** [28]			
T1–T2	1	-	
T3–T4	1.245	0.461–3.365	0.665
**Complications** [26]			
No-I–II	1	-	
III–IV	1.464	0.330–6.486	0.616

BMI: Body Mass Index.
